# Autochthonous Cases of Mucosal Leishmaniasis in Northeastern Italy: Clinical Management and Novel Treatment Approaches

**DOI:** 10.3390/microorganisms8040588

**Published:** 2020-04-18

**Authors:** Valeria Gaspari, Irene Zaghi, Giovanni Macrì, Annalisa Patrizi, Nunzio Salfi, Francesca Locatelli, Elena Carra, Maria Carla Re, Stefania Varani

**Affiliations:** 1 Unit of Dermatology, Head and Neck Department, St. Orsola Malpighi University Hospital, via Massarenti 9, 40138 Bologna, Italy; annalisa.patrizi@unibo.it; 2 Infectious Diseases Unit, Department of Medical and Surgical Sciences, University of Bologna, 40138 Bologna, Italy; irene.zaghi@gmail.com; 3 Unit of Otorhinolaryngology, Head and Neck Surgery and Audiology, St. Orsola-Malpighi University Hospital, 40138 Bologna, Italy; giovanni.macri@aosp.bo.it; 4 Department of Experimental, Diagnostic and Specialty Medicine, University of Bologna, 40138 Bologna, Italy; mariacarla.re@unibo.it (M.C.R.); stefania.varani@unibo.it (S.V.); 5 Pathology Unit, St. Orsola Malpighi University Hospital, 40138 Bologna, Italy; nunzio.salfi@aosp.bo.it (N.S.); francesca.locatelli@ausl.romagna.it (F.L.); 6 Istituto Zooprofilattico Sperimentale della Lombardia e dell’Emilia-Romagna “Bruno Ubertini”, 25124 Brescia, Italy; elena.carra@izsler.it; 7 Regional Reference Center for Microbiological Emergencies (CRREM), Unit of Microbiology, St. Orsola Malpighi University Hospital, 40138 Bologna, Italy

**Keywords:** head and neck mucosal leishmaniasis, novel ways of administration of anti-leishmanial drugs, endoscopic surgical treatment

## Abstract

Mucosal leishmaniasis (ML) is a rare clinical variant of tegumentary leishmaniasis in Mediterranean Europe. Here we report on three autochthonous cases of head and neck ML in patients living in Northeastern Italy. Patients presented with non-specific, long-standing symptoms of upper respiratory tract involvement, mimicking other diseases. Parasitological diagnosis was reached by histopathology, immunohistochemistry and molecular biology on tissue specimens. *Leishmania infantum* was identified by molecular typing in all three cases. All patients reached a complete remission with protracted multivalent antileishmanial drugs; in one case, a novel approach of combined medical and endoscopic surgical treatment was carried out. High clinical suspicion led to a prompt diagnosis and deployment of a multivalent treatment. ML should be considered in the differential diagnosis of nasal, oral, and pharyngolaryngeal lesions in endemic areas. A prompt diagnosis is mandatory to establish a correct management; different antileishmanial medications as well as endoscopic surgical options may be required to reach a complete remission.

## 1. Introduction

Mucosal leishmaniasis (ML) is a rare clinical variant of tegumentary leishmaniasis in Mediterranean Europe [1]. In France, ML represents around 2% of autochthonous *Leishmania* cases [2], while its prevalence in other countries is unknown. ML is mainly caused by *Leishmania infantum* (*L. infantum*) and is transmitted by the bite of sandflies of the genus *Phlebotomus*. Clinical manifestations of ML are variable, including nodules, polypoid lesions or granular inflammation and may involve the buccal area, pharyngeal and laryngeal regions and, less frequently, the nose [3,4,5,6,7].

Mediterranean ML is usually not associated with previous cutaneous lesions, which differentiates this tegumentary manifestation from the more common New World mucocutaneous leishmaniasis (NWML) [1,3]. Other differences are reported between NWML and Mediterranean ML; for example, *L. braziliensis* or *L. panamensis* are causing NWML, while *L. infantum* is the usual agent of ML, although *L. major* and *L. tropica* can also be involved [1]. Furthermore, the nasal cavity is commonly affected in NWML cases, but, less frequently in Mediterranean ML cases, and patients with ML acquired in the Mediterranean region have a better prognosis than those who acquired NWML in Latin America [3]. In NWML, destructive lesions with few parasites and high levels of tumor necrosis factor-α (TNF-α) have been reported [3]. This suggests that an uncontrolled immune response with increased production of pro-inflammatory cytokines is responsible for the tissue damage. Conversely, the pathogenesis of Mediterranean ML is still obscure. 

Despite the fact that there is no validated guideline for therapy of the rare ML in Mediterranean countries, miltefosine (50 mg tid × 28 days) and liposomal amphotericin B (L-Amb; 21–40 mg/kg total dose) are suggested as the first choices of treatment [4,7]. For NWML, pentavalent antimonials (Sb 20 mg/kg/day for 28–30 days) are still the gold standard of treatment, but also L-Amb (18–40 mg/kg total dose) can be used [1,3]. Pentoxyfilline (400 mg tid for 30 days) can be added to antimonials in NWML because of its capacity to downregulate TNF-α and inhibit leukocyte migration and adhesion [8,9]. Combining antimonials with pentoxifylline in NWML reduces the healing time significantly and prevents the need for further courses of Sb [10].

We recently observed an upsurge of visceral and cutaneous leishmaniasis in the Bologna province, Northeastern Italy [11,12]. Here, we report three cases of ML in patients living in this area.

## 2. Description of Cases

### 2.1. Case 1

**Figure 1:**
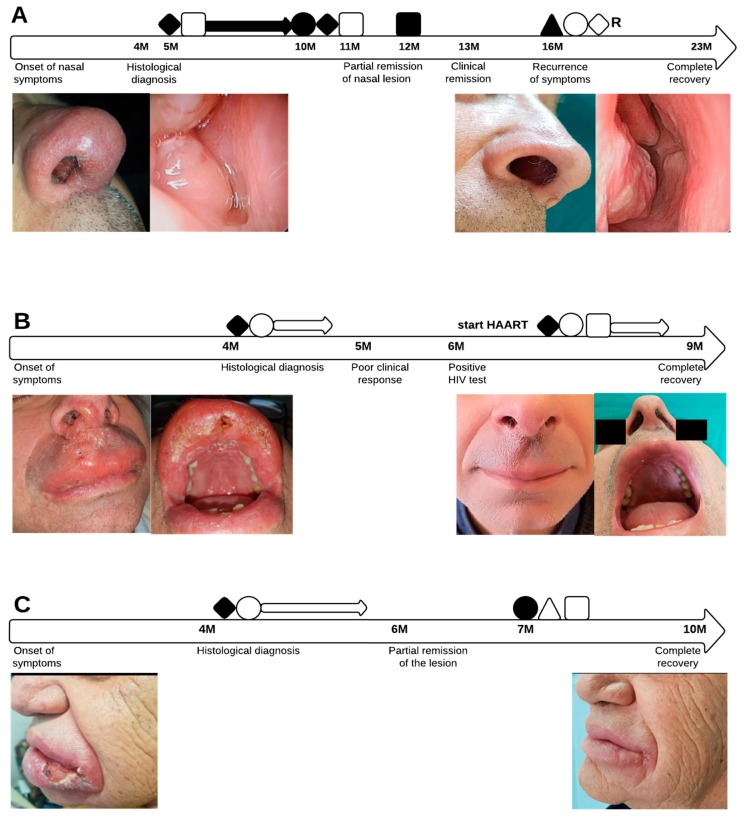
(**A**–**C**). Clinical course and treatment of mucosal leishmaniasis (ML) cases. (**A**) Case 1; clinical and endoscopic appearance of the nasal lesion before and after treatment. (**B**) Case 2; upper lip and nasal lesions before and after treatment. (**C**) Case 3; ulcerated lesion of the lower lip disappeared after treatment. ◆: intralesional meglumine antimoniate; ◊R: intralesional meglumine antimoniate via rhinoendoscopy; ●: miltefosine; o: pentamidine; ■: amphotericin B; □: oral fluconazole; ▲: surgical debridement; △: allopurinol.

**Figure 2:**
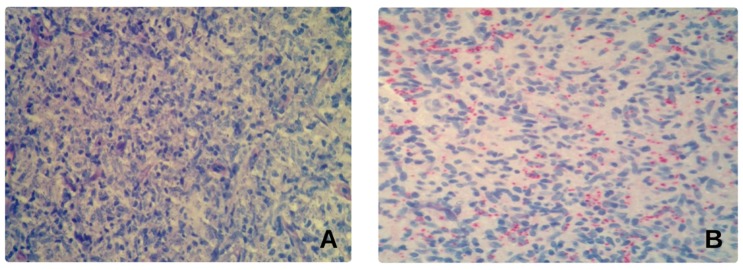
Histologic examination of ML bioptic specimen (Case 1). (**A**) Giemsa staining revealing a non-necrotizing granulomatous inflammatory infiltration, rich in plasma cells and epithelioid histiocytes containing eukaryotic elements, suspected for *Leishmania*. (**B**) Immunohistochemistry staining with CD1a confirmed an intense positivity for CD1a of *Leishmania* amastigotes in parasitized macrophages and in extracellular space.

A 63-year-old, immunocompetent man presented with rhinitis, mild epistaxis and nasal obstruction. Travel to Croatia was reported six months before symptoms onset. Physical examination revealed a mild erythema on the top of the nose and an ulcerated obstructive mass involving the right nostril and the vestibule extending to the anterior nasal valve. He was clinically diagnosed with nasal polyposis (Figure 1A) and biopsied. The biopsy specimen was formalin fixed, embedded in paraffin and stained with hematoxylin and eosin (H&E) and Giemsa; histology revealed suspected leishmanial bodies (Figure 2A). Immunohistochemistry was performed on an automated Immunostainer (Tucson, Arizona, USA) applying an anti-CD1a antibody (clone EP3622, CELL MARQUE); microscopic examination confirmed an intense positivity for CD1a within the *Leishmania* amastigotes (Figure 2B). Leishmanial DNA was also detected in the paraffined-embedded biopsy by using two in-house, real-time PCR assays that amplified a segment of the small-subunit rRNA gene of *Leishmania* [13] as well as a segment of the kinetoplast DNA [14], respectively, as previously described [12]. For species identification, a region of the Internal Transcribed Spacer-1 (ITS-1) was amplified and sequenced according to El Tai et al. [15] and following the protocols described by Rugna et al. [16]; ITS-1 typing displayed the presence of *L. infantum*. The presence of anti-*Leishmania* IgM and IgG antibodies was investigated by employing the Leishmania ELISA IgG + IgM kit (Vircell, Granada, Spain), but specific antibodies were not detected in the patient’s serum. Treatment was started with the association of intralesional meglumine antimoniate (one injection per week for four weeks) plus fluconazole (100 mg twice a day for one month). As the nasal mass only partially decreased, miltefosine (50 mg three times a day for one month) was added. After six months, the patient underwent a second endonasal biopsy, which revealed an extensive granulomatous process with the persistence of CD1a-positive amastigotes and leishmanial DNA. The patient was then started on IV L-Amb 3 mg/kg/day at day 1 to 5, 14 and 21 following international guidelines [1]. After an apparent remission, the patient returned with recurrence of the endonasal mass two months later; both *Leishmania* amastigotes and *Leishmania* DNA were detected in the biopsy specimen. After consultation with otorhinolaryngologists, the patient underwent a surgical protocol including surgical debulk of the macroscopic mass and intralesional meglumine antimoniate submucosal injections with a syringe of 1 mL and an insulin needle. All these passages were made under local anesthesia with the topical application of 2% Xylocaine (2% Lidocaine Hydrochloride, AstraZeneca, Sweden) through rhino-endoscopy to reach the upper part of the anterior nasal valve. Rigid nasal endoscopy was performed using a 30° 4 mm diameter rigid nasal endoscope (Karl Stortz Sinuscope, KARL STORZ GmbH & Co. KG, Tuttlingen, Germany). The patient underwent one injection of 1 mL of meglumine per week for two weeks, associated with pentamidine isethionate (3 mg/kg, weekly IM injections for two weeks). The surgical–medical combined therapy led to a rapid improvement of the patient’s clinical status (Figure 1A), allowing better air flow through the nasal vestibule obstructed by the thickened infected tissue. At a 12-month follow-up the patient exhibited a complete clinical and endoscopic recovery.

### 2.2. Case 2

A 59-year-old patient presented with a six-month history of painful nasal and oral bleeding and chewing difficulties. He reported not travelling abroad in the last two years. Clinical examination revealed an edematous infiltration of the upper lip and the anterior buccal mucosa and an ulcerated-crusted lesion in the right nasal vestibule (Figure 1B). Biopsy revealed a mixed inflammatory infiltrate and numerous amastigotes in both H&E and Giemsa stained sections, confirmed by CD1a immunohistochemistry. *Leishmania* DNA was detected on the tissue specimen by the two abovementioned real time PCR assays and results of ITS-1 typing indicated *L.infantum*. Antileishmanial therapy was started with intralesional meglumine antimoniate (1 mL per week), and pentamidine isethionate (3 mg/kg, weekly IM injections for three weeks), with little or no response. Risk factors for immunosuppression were investigated, including HIV infection. The HIV test turned positive with a CD4+ count of 200/mmc and a viral load of 234.995 copies/mL. Peripheral blood and bone marrow aspirate were examined and tested negative for leishmanial DNA, ruling out visceral involvement. The patient started antiretroviral therapy with the combination of tenofovir/emtricitabine and darunavir/cobicistat and was again put on treatment for ML with oral fluconazole (200 mg once a day), intralesional meglumine antimoniate (one injection/week) and injections of pentamidine isethionate (3 mg/kg, weekly IM injections) for three weeks, with complete remission of the lesions (Figure 1B) confirmed at a six-month follow-up.

### 2.3. Case 3

A 77-year-old immunocompetent man presented with a massive ulcerated bleeding lesion on the lower lip extending to the oral cavity mucosa (Figure 1C). He was a heavily smoker with no history of recent travel abroad; he only reported a traumatic cut in the lesioned area. In the suspicion of malignancy, the lesion was biopsied; histology revealed an intense chronic flogistic reaction and dysplastic aspects, consistent with a traumatic lesion. Owing to the persistence of symptoms, the patient was re-biopsied; specimens showed a mixed inflammatory infiltrate with leishmanial amastigotes detected by H&E and Giemsa staining. CD1a immunohistochemistry showed numerous *Leishmania* amastigotes, and leishmanial DNA was also detected in the biopsy specimen by the two PCR assays amplifying a fragment of the small-subunit rRNA gene as well as of the kinetoplast DNA, respectively. Molecular typing indicated the presence of *L. infantum*. The patient underwent a multivalent therapy, including cryotherapy, intralesional meglumine antimoniate (one injection/week) and IM pentamidine isethionate injections (one per week, 3 mg/kg for six weeks), with only partial remission. Subsequent dermoscopy still showed pathological aspects and therapy was modified to oral miltefosine 150 mg/day for one month, allopurinol 300 mg/day for three months and fluconazole 200 mg/day for three weeks [17,18,19], with complete remission at a 3-month follow-up (Figure 1C).

### 2.4. Ethics

All subjects gave their informed consent for inclusion before they participated in the study. The study was conducted in accordance with the Declaration of Helsinki, and the protocol was approved by the Ethics Committee of the St. Orsola-Malpighi University Hospital (prot. n.3175/2018, 29 October 2018).

## 3. Discussion

An elevated circulation of *Leishmania* has been recently observed in Northeastern Italy [11,12]; therefore, it is not surprising that autochthonous cases of ML have emerged in the same area.

Because of the long incubation period and time to diagnosis, it may be difficult to distinguish whether cases of tegumentary leishmaniasis are autochthonous or imported; therefore, accurate patient history and parasite identification are crucial [20]. A lack of travel history in areas that are endemic for NWML and species identification as *L. infantum* strongly suggests an autochthonous origin of the current ML cases. All three cases presented with long-standing mucosal disease, with a duration of around 6 months from the onset of symptoms to the diagnosis. Delayed diagnosis is a common feature of ML and NWML [2,21,22], which is often misdiagnosed as other upper respiratory tract diseases. Since the overlap in clinical presentation (ulcerated and bleeding masses) of ML and neoplasia, leishmaniasis should be taken into consideration in the differential diagnosis of head and neck cancer in patients from endemic areas or who report travelling to endemic countries.

The diagnosis of ML was performed by histology and molecular methods with detection of leishmanial amastigotes in tissue sections as well as leishmanial DNA. Detection of parasites in tissue sections may be difficult when only a few parasites are present; in these cases, CD1a staining can enhance the sensitivity of histological examination [23,24]. In Case 1, the diagnosis of ML was brought up by immunohistochemical staining, while regular H&E and Giemsa staining produced less clear results. The diagnosis was then confirmed by molecular methods; in our study, real-time PCR enabled confirmation of ML diagnosis in all three cases. Evidence suggests that PCR is a highly sensitive and specific method for detecting leishmanial DNA in mucosal biopsies [2,5,25]. In addition, in one out of three cases serology tested negative (Case 1), and this is compatible with a low titer of circulating antibodies against *Leishmania* in ML [26].

At present, there is no standardized treatment for ML; the drug, dosage and duration of therapy should be individualized for each case, considering the clinical aspect of the lesions, the infecting *Leishmania* species and the immunological status of the patient. Specific treatment regimens are mainly guided by practical considerations and the personal experience of the treating physician [3,7]. A systemic treatment is considered mandatory to prevent morbidity (e.g., disfigurement) and mortality (e.g., aspiration pneumonia and respiratory obstruction). The examined patients reached complete remission by combining multivalent systemic and local treatment; drugs with distinct efficacy against *Leishmania* were used, including those targeting ergosterol biosynthesis (fluconazole), hampering thiol metabolism thereby inducing DNA fragmentation (meglumine antimoniate) and hindering DNA synthesis at mitochondrial level (pentamidine) [27]. 

Furthermore, in Case 1, a novel approach of combined medical and endoscopic treatment was successfully carried out; meglumine antimoniate injections through rhino-endoscopy were combined with surgical endoscopic debridement, which allowed coverage of all the internal areas of the lesion up to the nasal valve. To the best of our knowledge, this is the first ML case successfully treated with endonasal intralesional injection by rhino-endoscopy.

ML is often detected in immunocompromised individuals [1] presenting with extensive lesions and poor response to antiparasitic therapy. In Case 2, the remission was likely due to the introduction of antiretroviral therapy in combination with a multivalent antileishmanial treatment.

## 4. Conclusions

In conclusion, ML should be considered in the differential diagnosis of granulomatous plaques and nodules of the head and neck mucosa in immunocompetent and immunocompromised individuals living in endemic areas for leishmaniasis or reporting travels to endemic areas.

Combined dermatological and otolaryngologist examination is important for the clinical management of ML [28]. A biopsy of the lesion is mandatory to perform a parasitological diagnosis, thus allowing the prompt introduction of antileishmanial therapy. In some cases, as in Case 1 of this report, it may be necessary to introduce different methods of drug administration.
